# Distribution and Surgical Treatment of Corneal Dystrophies Over Eight Decades (1945–2024): An Analysis of Histopathologically Confirmed Cases from a German Center

**DOI:** 10.1007/s44197-025-00458-y

**Published:** 2025-08-28

**Authors:** Othmane Touirssa, Philip Maier, Daniel Boehringer, Claudia Auw-Haedrich, Mateusz Glegola, Thomas Reinhard, Simone Nuessle

**Affiliations:** 1 Eye Center, University Medical Center Freiburg, Albert-Ludwigs-University of Freiburg, Killianstrasse 5, 79106 Freiburg, Germany; 2 Present Address: Eye Clinic, University Hospital Basel, Mittlere Str. 91, Basel, 4056 Switzerland

**Keywords:** Cornea, Corneal dystrophies, Keratoplasty, Transplantation, Surgical trends, Histopathology, Ophthalmic pathology, Longitudinal study, Ocular epidemiology

## Abstract

**Background:**

Corneal dystrophies are inherited disorders that can lead to significant visual impairment and often require surgical intervention in advanced stages. Fuchs endothelial corneal dystrophy (FECD) is the most frequently diagnosed type in Western countries and remains a leading global indication for corneal transplantation. In contrast, non-Fuchs dystrophies represent a diverse group of less common entities, each with distinct clinical features, surgical considerations, and regional variations in incidence and management. Despite their relevance, long-term data on the full spectrum of corneal dystrophies remain scarce. This study aimed to evaluate the distribution and temporal trends in dystrophy types and associated surgical procedures over eight decades at a tertiary referral center in Germany.

**Methods:**

This retrospective analysis included 3 827 histopathologically confirmed corneal dystrophy specimens identified from an archive of 58 150 ophthalmic specimens collected between 1945 and 2024. Extracted data included dystrophy type, patient age at surgery, sex assigned at birth and associated surgical procedures. Distribution and temporal trends were analyzed descriptively.

**Results:**

FECD accounted for 90.3% (*n* = 3 455) of all cases, with a more than 15-fold increase in annual cases between 2003 and 2024. Its surgical management transitioned from exclusive use of penetrating keratoplasty (PKP) to posterior lamellar keratoplasty in over 99% of cases by 2024. Among non-Fuchs dystrophies (*n* = 372), granular (21.2%), macular (17.5%), and lattice dystrophy (17.2%) were most frequent. These exhibited greater surgical variability, reflecting their heterogeneity across 21 non-Fuchs dystrophy types in this study. Stromal and epithelial-stromal dystrophies were predominantly managed with PKP, whereas superficial epithelial and basement membrane dystrophies were increasingly treated with phototherapeutic or manual superficial keratectomy. Limbo-keratoplasty was introduced in the early 2000s for recurrent subepithelial and epithelial-stromal types.

**Conclusion:**

This study provides unique insights into the type distribution and surgical management of corneal dystrophies over eight decades in a German center, encompassing nearly all IC3D-classified entities. The marked increase in FECD specimen numbers and the shift toward lamellar keratoplasty reflect evolving clinical practices and rising demand on corneal transplantation services. The broader clinical spectrum and procedural diversity among non-Fuchs dystrophies underscore the ongoing need for pathology-specific management strategies tailored to population-specific needs.

## Background

Corneal dystrophies are a heterogeneous group of primary, inherited disorders characterized by abnormal deposits within specific corneal layers and resulting in varying degrees of visual impairment [1, 2]. They are classified based on their initial anatomical location (epithelial, stromal, or endothelial) as well as clinical, genetic, and histopathological features, according to the IC3D classification (International Committee for the Classification of Corneal Dystrophies) [3]. While Fuchs endothelial corneal dystrophy (FECD) is the most common type and a leading indication for corneal transplantation, the broader spectrum of non-Fuchs dystrophies presents with substantial regional and clinical variability [1–7].

Surgical approaches for corneal dystrophies have evolved substantially over recent decades. The historical standard, penetrating keratoplasty (PKP), has been increasingly supplanted by lamellar techniques like Descemet membrane endothelial keratoplasty (DMEK) for endothelial disease and phototherapeutic keratectomy (PTK) for superficial dystrophies, which are preferred for their ability to target specific corneal layers and tissue-sparing approach, offering faster recovery times [8–10].

This rapid evolution in surgical care exists within the context of a profound global mismatch between donor tissue supply and patient demand, making the efficient allocation of resources paramount [4]. This imbalance underscores the importance of understanding not only the epidemiology of corneal diseases but also regional trends in surgical indications to inform resource planning and health policy.

Despite the impact of corneal dystrophies on surgical demand, healthcare resources, patient quality of life [11–13] and also the central role of transplantation, most existing studies remain limited in scope, often focusing on individual dystrophy types or covering only short observational periods. As a result, longitudinal trends in the distribution of corneal dystrophy types and associated surgical procedures remain insufficiently characterized in the literature, limiting the evidence base for population-specific healthcare planning.

This study retrospectively analyzed all histopathologically confirmed corneal dystrophy specimens collected from 1945 to 2024 at a German tertiary referral center, offering a unique longitudinal perspective on the evolving landscape of corneal dystrophies and their surgical management within a defined population. The objectives were to describe the distribution and temporal trends of dystrophy types, assess demographic characteristics, and examine temporal changes in surgical techniques, as well as the distribution of surgical procedures for each dystrophy type.

## Methods

This retrospective observational study was conducted at the ophthalmic pathology laboratory of the Eye Center at Medical Center, University of Freiburg. The analysis involved ophthalmic pathology reports of all histological samples archived between 1945 and 2024, comprising specimens collected from the Eye Center but also specimens submitted by external clinics or institutions. This study adhered to the Declaration of Helsinki. The study was approved and the need for informed consent was waived by the local ethics committee (Ethics Committee at the University of Freiburg Medical Center, approval number 25-1067-S1-retro).

Over the course of eight decades, specimens were analyzed by different experienced ophthalmic pathologists who were/are also eye surgeons themselves, using light microscopy. Standard hematoxylin and eosin (H&E) and Periodic acid–Schiff (PAS) staining was performed on all specimens. Based on morphological findings and clinical suspicion, diagnoses were confirmed with special stains when necessary, while electron microscopy was utilized in select cases where it was required for a definitive diagnosis. Clinical-pathological correlations were facilitated using available clinical data, photographs, and surgeon-provided descriptions.

All specimens received by our laboratory between 1945 and 2024 were analyzed. Specimens were included in this study if they were diagnosed histopathologically as a corneal dystrophy, regardless of the initial clinical suspicion. Historical diagnostic terms were retrospectively updated in accordance with the 3rd Edition of the IC3D classification [3] by a physician with expertise in ophthalmic pathology, ensuring consistency across the archival dataset and alignment with current nomenclature. Cases described only as “consistent with” corneal dystrophy were excluded. Cases diagnosed histopathologically as corneal dystrophies but with ambiguous histopathological diagnostic information were categorized based on the dystrophy’s location into unspecified epithelial, epithelial-stromal, stromal, endothelial corneal dystrophy, or, in rare cases, unspecified localization and type. Cases initially labeled as granular corneal dystrophy without distinction between type 1 and type 2 were classified as unspecified granular dystrophy.

For each included specimen, data were extracted from histopathology reports, pathology request forms, and any accompanying clinical documentation. The recorded variables included patient demographics (age at surgery, sex assigned at birth) and the surgical procedure. Surgical interventions were classified as PKP, limbo-keratoplasty (LimboKP), posterior lamellar keratoplasty, manual superficial keratectomy (MSK), PTK, or enucleation. To ensure consistent classification, Descemet Stripping Automated Endothelial Keratoplasty (DSAEK) cases for FECD were grouped with DMEK under ‘posterior lamellar keratoplasty’ due to occasional ambiguity in operative reports. For non-Fuchs dystrophies, all posterior lamellar keratoplasty cases were treated with DMEK. All data were systematically entered into a spreadsheet database (Excel) for analysis. Cases with incomplete demographic data were flagged but retained for analysis, as all had a confirmed histopathological diagnosis.

Descriptive statistics were calculated for demographic data like age at surgery and sex assigned at birth, dystrophy type distributions and surgical procedures. Continuous variables were reported as means with standard deviations or medians with interquartile ranges (IQR), depending on their distribution as assessed by the Shapiro-Wilk test. Categorical variables were summarized as counts and percentages. Data visualization and statistical analyses were performed using Python (Version 3.12.3) within the IDLE environment (Integrated Development and Learning Environment) on macOS.

## Results

Between 1945 and 2024, a total of 3 827 specimens were diagnosed as corneal dystrophies from an archive of 58 150 specimens. Of these, 3 455 (90.3%) were classified as FECD, and 372 (9.7%) were diagnosed as non-Fuchs corneal dystrophies.

### Demographic Characteristics

#### Sex Assigned at Birth and Age Distribution

As shown in Table 1, among FECD specimens with recorded sex (*n* = 2,973), females accounted for 59.6% (*n* = 1,772), while males accounted for 40.4% (*n* = 1,201). The mean age of FECD patients was 70.1 years (± 9.68), with a median of 71.0 years (IQR 13).

**Table 1:** Comprehensive overview of age at first surgery, youngest age, sex assigned at birth, specimen and patient data in Fuchs and non-Fuchs corneal dystrophies

Dystrophy Type	Total Specimens (% of NFD)	Total Patients (% of NFD)	Sex Distribution (♀ / ♂)	Median Age in years (IQR)	Youngest Age in years
**Fuchs endothelial dystrophy** (FECD)	3455	Not applicable	1772 (59.6%) / 1201 (40.4%) †	71 (13) ‡	25
**Non-Fuchs Dystrophies** §					
**Epithelial and Subepithelial Dystrophies**					
EBMD	25 (6.72%)	25 (8.99%)	9 (36%) / 16 (64%)	54 (18.25)	38
ERED	2 (0.54%)	2 (0.72%)	1 (50%) / 1 (50%)	57	57
Franceschetti (FRCD)	2 (0.54%)	1 (0.36%)	0 (0%) / 1 (100%)	75	75
SMCD	5 (1.34%)	5 (1.80%)	3 (60%) / 2 (40%)	25 (32)	17
Meesmann (MECD)	5 (1.34%)	2 (0.72%)	1 (50%) / 1 (50%)	46	40
Lisch (LECD)	9 (2.42%)	6 (2.16%)	2 (33.3%) / 4 (66.7%)	53.5 (16.25)	32
Gelatinous (GDLD)	29 (7.80%)	6 (2.16%)	5 (83.3%) / 1 (16.7%)	34.5 (22.25)	19
**Epithelial-Stromal TGFBI Dystrophies**					
Reis–Bücklers (RBCD)	4 (1.08%)	4 (1.44%)	3 (75%) / 1 (25%)	53	45
Thiel–Behnke (TBCD)	15 (4.03%)	11 (3.96%)	6 (54.5%) / 5 (45.5%)	38 (29.5)	22
Lattice (LCD) § ¶	64 (17.20%)	44 (15.83%)	22 (52.4%) / 20 (47.6%)	54.5 (21.75)	23
Granular, unspecified type (GCD)	25 (6.72%)	23 (8.27%)	13 (56.5%) / 10 (43.5%)	49 (19.5)	16
Granular type 1 (GCD1)	36 (9.68%)	24 (8.63%)	10 (41.7%) / 14 (58.3%)	54.5 (25)	16
Granular type 2 (GCD2)	18 (4.84%)	16 (5.76%)	7 (43.8%) / 9 (56.2%)	59.5 (18.75)	17
“Gly623Asp”-Mutation ¶	3 (0.81%)	1 (0.36%)	0 (0%) / 1 (100%)	54	54
**Stromal Dystrophies**					
Macular (MCD)	65 (17.47%)	49 (17.63%)	24 (49%) / 25 (51%)	37 (29)	8
Schnyder (SCD)	11 (2.96%)	10 (3.60%)	4 (40%) / 6 (60%)	53 (12)	37
Congenital Stromal (CSCD)	4 (1.08%)	3 (1.08%)	2 (66.7%) / 1 (33.3%)	8	5
PACD	1 (0.27%)	1 (0.36%)	0 (0%) / 1 (100%)	51	51
Pre-Descemet (PDCD)	1 (0.27%)	1 (0.36%)	0 (0%) / 1 (100%)	49	49
**Endothelial Dystrophies**					
PPCD	12 (3.23%)	10 (3.60%)	7 (70%) / 3 (30%)	29.5 (31)	7
CHED	2 (0.54%)	2 (0.72%)	0 (0%) / 2 (100%)	21.5	4
**Unspecified Dystrophies**					
Epithelial dystrophy	7 (1.88%)	6 (2.16%)	3 (50%) / 3 (50%)	49.5 (35.5)	2
Epithelial-stromal dystrophy ¶	7 (1.88%)	7 (2.52%)	5 (71.4%) / 2 (28.6%)	68.5 (42.5)	5
Stromal dystrophy	2 (0.54%)	1 (0.36%)	1 (100%) / 0 (0%)	49	49
Endothelial dystrophy	16 (4.30%)	16 (5.76%)	8 (50%) / 8 (50%)	53.5 (28.75)	5
Unspecified localisation	2 (0.54%)	2 (0.72%)	0 (0%) / 2 (100%)	33	17
**Total of Non-Fuchs Dystrophies**	372	278	136 (49.3%) / 140 (50.7%)		

IQR, Interquartile range, calculated only if more than five patients; NFD, non-Fuchs corneal dystrophies; FECD, Fuchs endothelial corneal dystrophy; EBMD, Epithelial basement membrane dystrophy; ERED, Epithelial recurrent erosion dystrophy; FRCD, Franceschetti corneal dystrophy; SMCD, Subepithelial mucinous corneal dystrophy; MECD, Meesmann corneal dystrophy; LECD, Lisch epithelial corneal dystrophy; GDLD, Gelatinous drop-like corneal dystrophy; TGFBI, Transforming growth factor, beta-induced; RBCD, Reis–Bücklers corneal dystrophy; TBCD, Thiel–Behnke corneal dystrophy; LCD, Lattice corneal dystrophy; GCD, Granular corneal dystrophy – unspecified type; GCD1 Granular corneal dystrophy, type 1; GCD2 Granular corneal dystrophy, type 2; Gly, Glycin; Asp, Aspartic acid amino acid; Gly623Asp, corneal dystrophy associated with missense mutation in exon 14 of the TGFBI gene (G->A transition at nucleotide 1915) replacing glycin by aspartic acid amino acid at position 623; MCD, Macular corneal dystrophy; SCD, Schnyder corneal dystrophy; CSCD, Congenital stromal corneal dystrophy; PACD, Posterior amorphous corneal dystrophy; PDCD, Pre-Descemet corneal dystrophy; PPCD, Posterior polymorphous corneal dystrophy; CHED, Congenital hereditary endothelial dystrophy; IC3D Classification nomenclature was applied – Edition 3 - (3)

Youngest Age in years: the age at which the first specimen was obtained for each dystrophy type; ♀ Female; ♂ Male; † 2,973 among FECD specimens with recorded sex; ‡ Median age calculated based on age of patient associated to each specimen; § Sex information missing for 2 patients. ¶ Three specimens (two originally diagnosed as lattice dystrophy and one as unspecified epithelial-stromal dystrophy) were later reclassified as Gly623Asp mutation variants. For consistency, they are presented in the table under their original diagnostic categories

In non-Fuchs dystrophies, 278 patients contributed a total of 372 specimens. Sex distribution was nearly equal, with females accounting for 49.3% (*n* = 136) and males for 50.7% (*n* = 140), while sex data were unavailable for two patients.

The age at which the first specimen was obtained varied across dystrophy types (Table 1). The youngest median age was observed in patients with congenital stromal corneal dystrophy (CSCD) and congenital hereditary endothelial dystrophy (CHED). Among the most frequently diagnosed non-Fuchs dystrophies, patients with macular dystrophy (MCD) had a lower median age at surgery compared to those with granular (GCD) and lattice (LCD) dystrophies. A detailed summary of the age distribution for all recorded dystrophy types is provided in Table 1.

### Type-Specific Distribution of Corneal Dystrophies

In terms of anatomical classification, most specimens were endothelial dystrophies (*n* = 3485), comprising 3455 cases of Fuchs dystrophy and 30 cases of non-Fuchs endothelial dystrophies. These were followed by epithelial-stromal TGFBI dystrophies (*n* = 172), with stromal (*n* = 84) and epithelial/subepithelial dystrophies (*n* = 84) being the least common anatomic categories.

Among non-Fuchs dystrophies, granular dystrophies (GCD, all types combined; *n* = 79, 21.24%), MCD (*n* = 65, 17.47%) and LCD (*n* = 64, 17.20%) were the most frequently diagnosed dystrophy types. Among epithelial and subepithelial dystrophies, gelatinous drop-like dystrophy (GDLD; *n* = 29, 7.80%) and epithelial basement membrane dystrophy (EBMD; *n* = 25, 6.72%) were the most common. Stromal dystrophies were less common, with Schnyder corneal dystrophy (SCD; *n* = 11, 2.96%) being the most notable. Similarly, non-Fuchs endothelial dystrophies were infrequent, led by posterior polymorphous corneal dystrophy (PPCD; *n* = 12, 3.23%). A number of cases were categorized as unspecified dystrophies with certain localization but no defined type, reflecting diagnostic challenges in some specimens. A comprehensive breakdown of all dystrophy types, including their absolute counts and relative frequencies, is detailed in Table 1.

### Temporal Distribution and Trends of Fuchs and Non-Fuchs Dystrophies

**Fig. 1:**
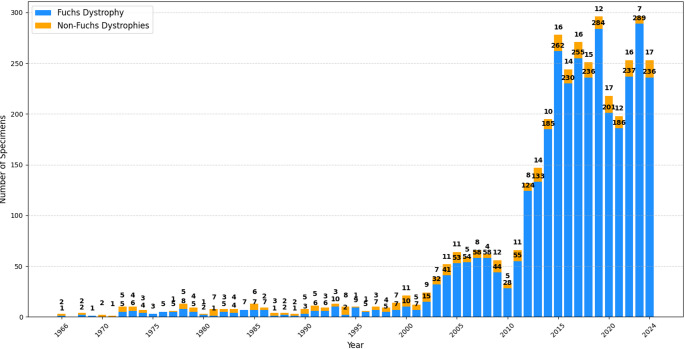
Annual distribution of histopathologically confirmed Fuchs (*n* = 3455) and non-Fuchs corneal dystrophies (*n* = 372) No corneal dystrophies were diagnosed between 1945 and 1965, although corneal specimens from that period exist in the archive. For this reason, the x-axis begins in 1966

No corneal dystrophies were diagnosed between 1945 and 1965 although corneal specimens from that period exist in the archive, with the first three cases diagnosed in 1966. The temporal analysis of corneal dystrophy specimens from 1966 to 2024 highlights a significant increase in the histopathological diagnosis of both Fuchs and non-Fuchs dystrophies over time (Fig. 1). FECD exhibited a particularly sharp rise in the number of specimens starting in the early 2000s, more than doubling between 2002 and 2003 (2002: 15 specimens, 2003: 32 specimens). This upward trend continued until 2008, reaching 62 specimens in this year. A slight decline was observed in 2009 and 2010 (56 and 33 specimens, respectively), after which the number of cases rose markedly throughout the 2010s (2011: 55 specimens, 2012: 124 specimens), peaking in 2019 with 296 specimens. A temporary decline was observed in 2020 and 2021 (218 and 198 specimens, respectively), before increasing again and ultimately reaching 236 specimens in 2024. Between 1966 and 2001, diagnoses of Fuchs and non-Fuchs dystrophies were relatively balanced. However, starting in 2002 (15 FECD specimens vs. 9 non-Fuchs specimens), non-Fuchs dystrophies became consistently less frequent compared to FECD. Despite this shift, the number of non-Fuchs dystrophy diagnoses continued to increase over time. Between 1966 and 1999, annual diagnoses of non-Fuchs dystrophies ranged from 0 to 8 specimens; between 2000 and 2010, they ranged from 4 to 12 specimens per year; and between 2011 and 2024, they ranged from 7 to 17 specimens annually.

**Fig. 2:**
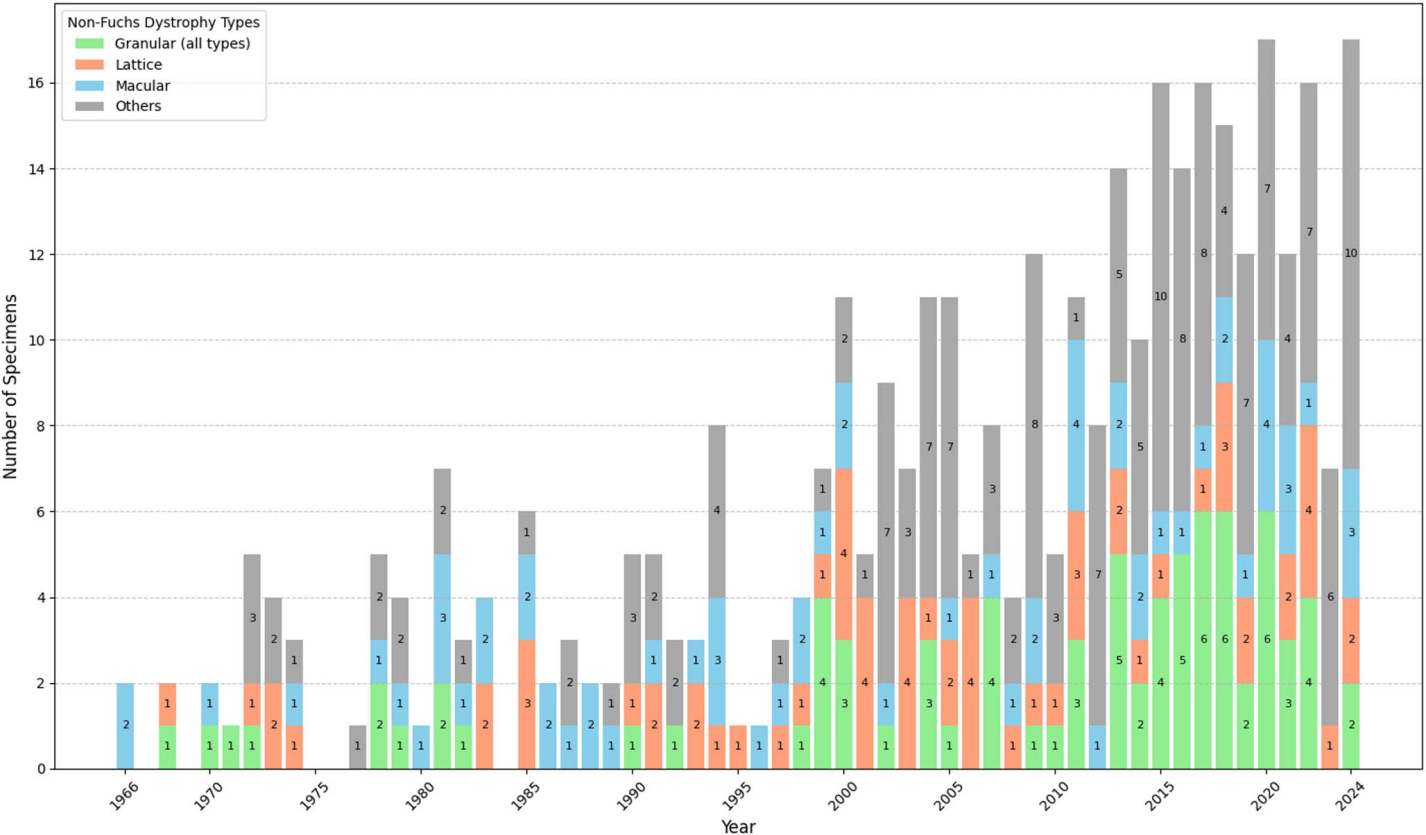
Annual distribution of histopathologically confirmed non-Fuchs dystrophies (*n* = 372) Dystrophies are grouped into four categories. Granular (all types): Granular corneal dystrophy (*n* = 79), all types; Lattice: Lattice corneal dystrophy (*n* = 64); Macular: Macular corneal dystrophy (*n* = 65); Others: other less frequent non-Fuchs corneal dystrophies (*n* = 164)

The temporal distribution of non-Fuchs dystrophies, illustrated in Fig. 2, reveals a gradual yet consistent increase in the histopathological diagnoses of macular, granular, and lattice dystrophies, which collectively dominate the spectrum of non-Fuchs corneal dystrophies (Fig. 2; Table 1). A slight increase in granular dystrophy specimens was observed starting in 1999, transitioning from a maximum of 2 specimens per year to up to 6 specimens annually. Diagnoses of MCD were consistently observed across the analyzed period, with up to 4 specimens reported annually. LCD diagnoses were evenly distributed across the timeline, with a slight increase in the two last decades, with a maximum of 4 specimens reported annually. Additionally, a more pronounced increase in the diagnoses of other, rarer non-Fuchs dystrophies was observed. Before 2002, annual diagnoses of these dystrophies did not exceed 4 specimens, whereas from 2002 onward, they increased to a maximum of 10 specimens per year.

### Surgical Interventions

#### Temporal Trends in Surgical Procedures

**Fig. 3:**
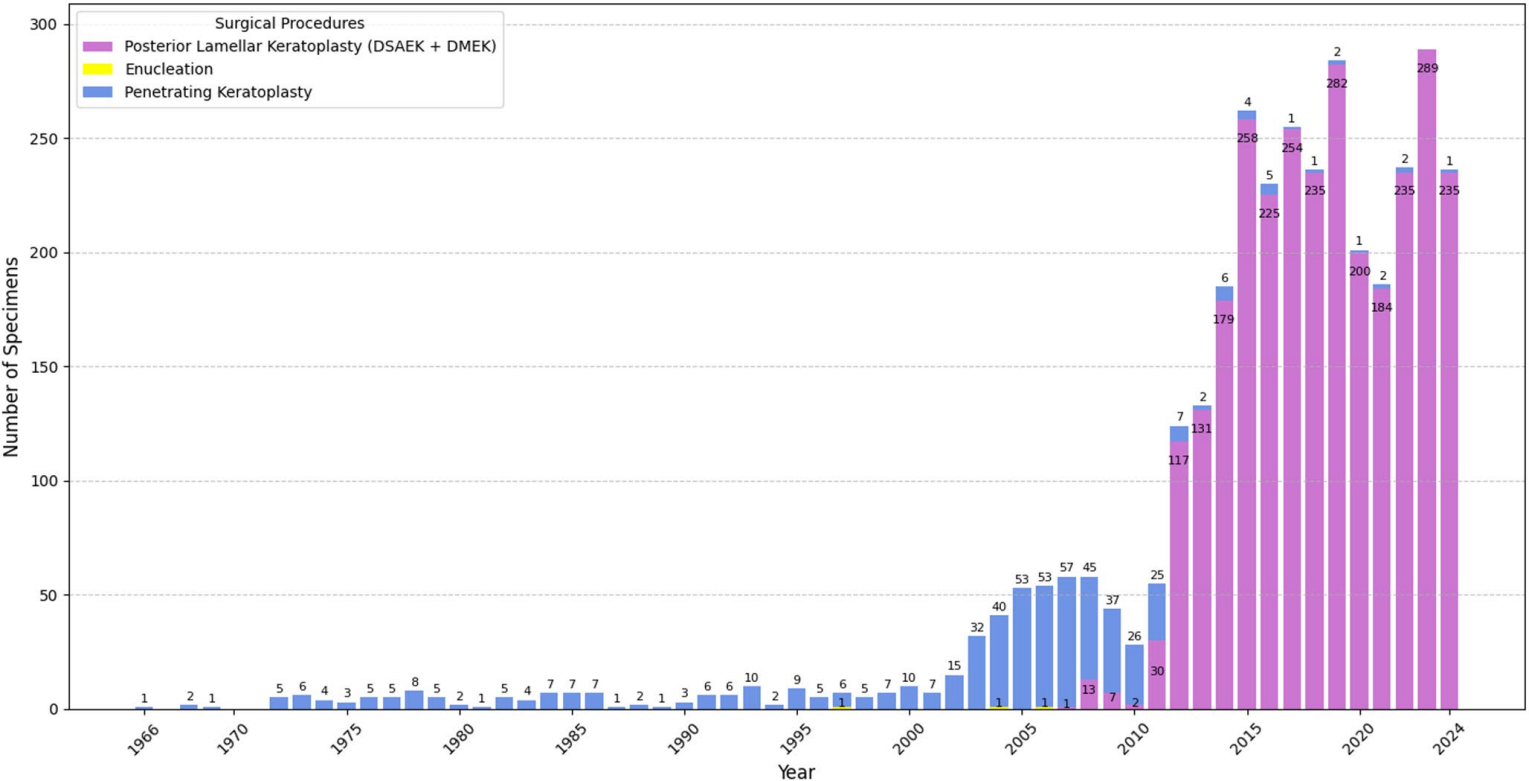
Temporal trends in surgical procedures for Fuchs Endothelial Corneal Dystrophy specimens (*n* = 3455) Annual distribution of surgical procedures associated with histopathologically confirmed Fuchs endothelial corneal dystrophy specimens. Posterior Lamellar Keratoplasty: Descemet stripping endothelial keratoplasty (DSAEK) and Descemet membrane endothelial keratoplasty (DMEK); Enucleation: enucleated eyes submitted postmortem or from living patients, for comprehensive histopathological examination, including corneal evaluation

The surgical management of FECD evolved significantly over the study period (Fig. 3). Initially, PKP was the predominant surgical method, accounting for all specimens prior to 2007, with the exception of rare instances of enucleation (three specimens in total) recorded as incidental findings unrelated to the primary management of FECD. From the late 2000s onwards, the use of posterior lamellar keratoplasty techniques gradually increased. By 2012, a marked shift in surgical practice was observed, with posterior lamellar keratoplasty accounting for 94.3% of specimens, and by 2024, it had largely supplanted PKP, with over 99.6% of specimens obtained through this technique.

**Fig. 4:**
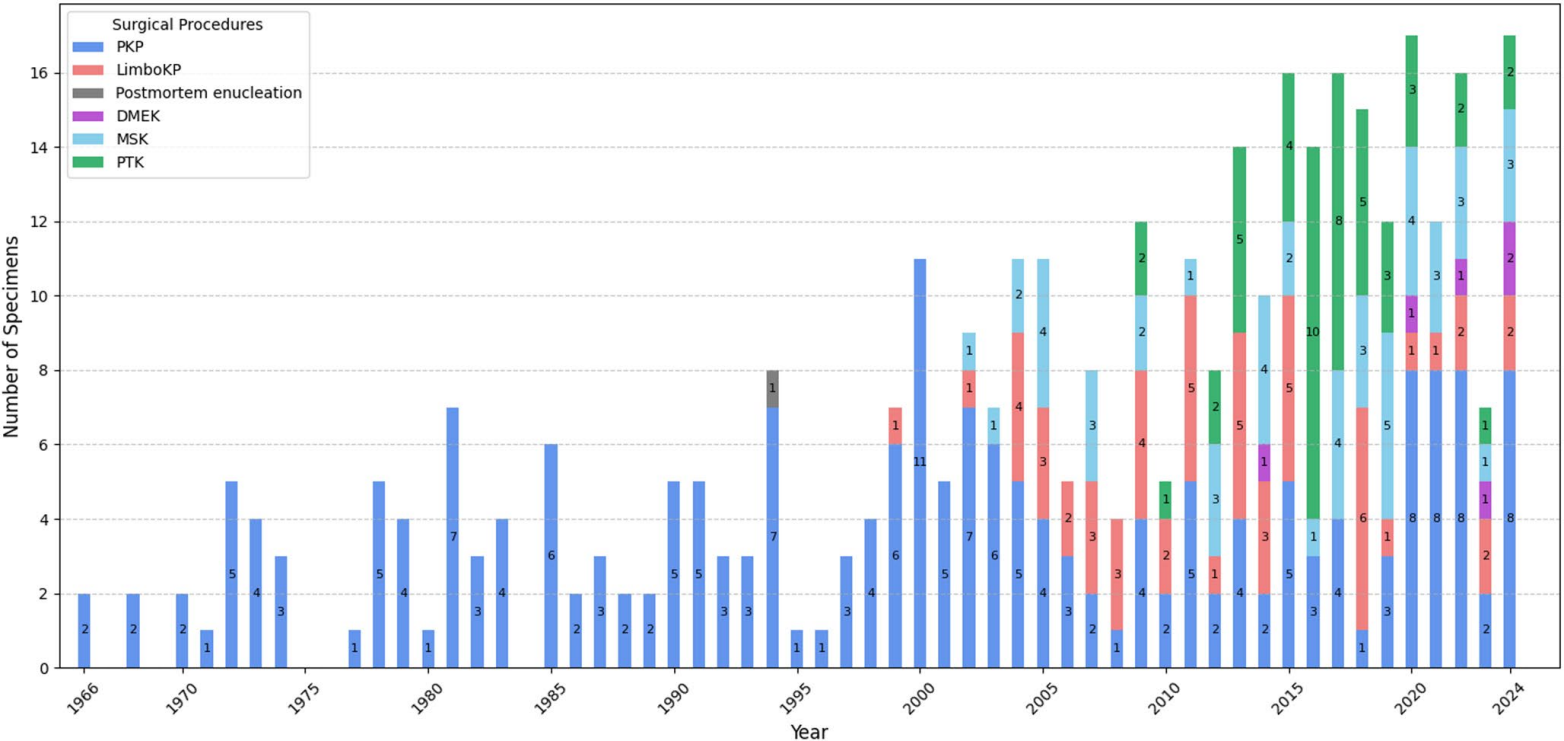
Temporal trends in surgical procedures for non-Fuchs dystrophy specimens (*n* = 372) Annual distribution of surgical procedures associated with histopathologically confirmed non-Fuchs corneal dystrophy specimens. PKP: penetrating keratoplasty; LimboKP: limbo-keratoplasty; Postmortem enucleation: the entire eye was obtained postmortem for histopathological examination; DMEK: descemet membrane endothelial keratoplasty; MSK: manual superficial keratectomy; PTK: phototherapeutic keratectomy

**Fig. 5:**
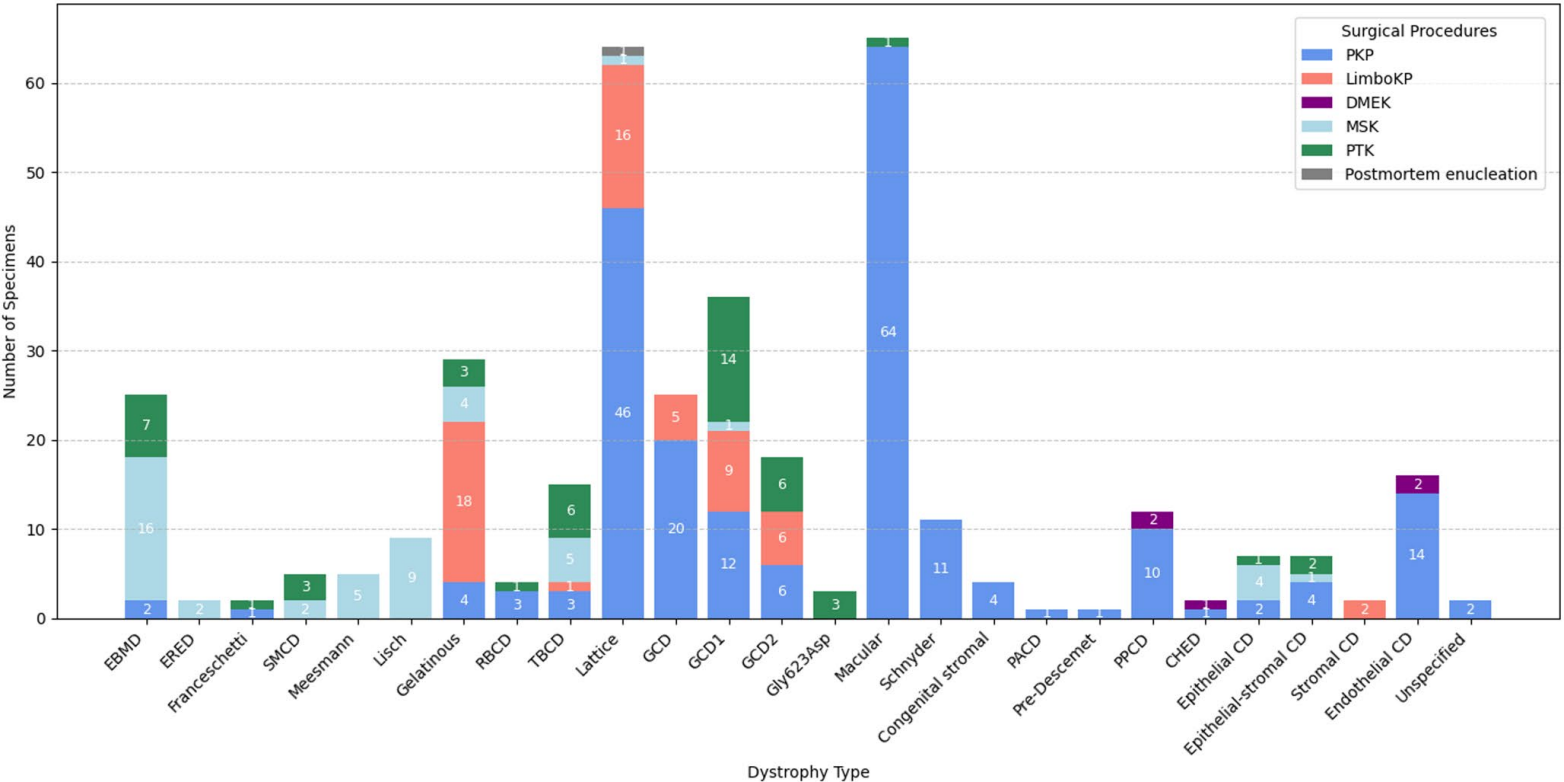
Distribution of surgical procedures by dystrophy type for non-Fuchs dystrophy specimens (*n* = 372) Overview of surgical procedures associated with each histopathologically confirmed corneal dystrophy type. PKP: penetrating keratoplasty; LimboKP: limbo-keratoplasty; DMEK: descemet membrane endothelial keratoplasty; MSK: manual superficial keratectomy; PTK: phototherapeutic keratectomy; Postmortem enucleation: the entire eye was obtained postmortem for histopathological examination; EBMD: Epithelial basement membrane dystrophy; ERED: Epithelial recurrent erosion dystrophy; Franceschetti: Franceschetti corneal dystrophy; SMCD: Subepithelial mucinous corneal dystrophy; Meesmann: Meesmann corneal dystrophy; Lisch: Lisch epithelial corneal dystrophy; Gelatinous: Gelatinous drop-like corneal dystrophy; RBCD: Reis–Bücklers corneal dystrophy; TBCD: Thiel–Behnke corneal dystrophy; Lattice: Lattice corneal dystrophy; GCD: Granular corneal dystrophy – unspecified type; GCD1: Granular corneal dystrophy, type 1; GCD2: Granular corneal dystrophy, type 2; Gly, Glycin; Asp, Aspartic acid amino acid; Gly623Asp: corneal dystrophy associated with missense mutation in exon 14 of the TGFBI gene (G->A transition at nucleotide 1915) replacing glycin by aspartic acid amino acid at position 623; Macular: Macular corneal dystrophy; Schnyder: Schnyder corneal dystrophy; CSCD: Congenital stromal, Congenital stromal corneal dystrophy; PACD: Posterior amorphous corneal dystrophy; Pre-Descemet: Pre-Descemet corneal dystrophy; PPCD: Posterior polymorphous corneal dystrophy; CHED: Congenital hereditary endothelial dystrophy; Epithelial CD: unspecified epithelial corneal dystrophy; Epithelial-stromal CD: unspecified epithelial-stromal corneal dystrophy; Stromal CD: unspecified stromal corneal dystrophy; Endothelial CD: unspecified endothelial corneal dystrophy; Unspecified: localization and type of the dystrophy not specified. Three specimens initially treated with PKP (two diagnosed as lattice dystrophy and one as an unspecified epithelial-stromal dystrophy) were later reclassified as Gly623Asp mutation variants. For consistency, they are presented in the figure under their original diagnostic categories

The surgical approaches for non-Fuchs dystrophies demonstrated notable diversity, reflecting the heterogeneity of these conditions (Fig. 4). PKP remained the predominant method throughout the study period, accounting for the majority of specimens (210/372, 56.45%), particularly for stromal dystrophies such as macular, lattice, and granular dystrophies (see Fig. 5 and *Distribution of surgical procedures*).

A notable shift occurred after 2003 with the growing adoption of LimboKP. Interestingly, two earlier LimboKP cases were documented, one from 1999 and another from 2002, both submitted by an external sender. From 2003 onward, the number of LimboKP cases gradually increased, reaching a peak of 6 annually in 2018. However, after 2018, a decline was observed, with annual cases decreasing to a maximum of 2 specimens per year between 2019 and 2024. Concurrently, a marked increase in specimens associated with MSK was observed from 2002 onward, primarily for the management of epithelial and subepithelial dystrophies. The number of MSK-related specimens remained relatively stable over time, with a maximum of 4 per year.

The first specimen of corneal dystrophy linked to PTK procedure for corneal dystrophy in this study was documented in 2009. The frequency of PTK procedures varied over time, peaking at 10 cases in 2016, followed by a subsequent decline in later years.

Five cases of non-Fuchs endothelial dystrophies were treated with DMEK.

#### Distribution of Surgical Procedures

The surgical management of non-Fuchs corneal dystrophies demonstrates significant variation, shaped by the anatomical localization and pathological mechanisms of each dystrophy type (Fig. 5).

PKP was the predominant surgical method, particularly for dystrophies affecting the deeper corneal layers. It was used in the vast majority of MCD cases (64/65 specimens) and was the primary approach for LCD (46/63) and GCD (38/79). Furthermore, PKP was the exclusive procedure documented for Schnyder and congenital stromal dystrophy. Even for non-Fuchs endothelial dystrophies like PPCD, PKP remained the most common intervention (10/12), with DMEK being used selectively in only five cases across all non-Fuchs endothelial types.

For superficial conditions, MSK was the primary treatment for epithelial dystrophies such as EBMD (16/25). PTK was also utilized for various epithelial and subepithelial dystrophies and was notably further utilized for granular dystrophies (20/79) when the superficial anterior stroma was primarily affected. Additionally, PTK was also performed in all three cases of corneal dystrophy associated with the Gly623Asp mutation of the TGFbeta-induced gene, a distinct subtype characterized by amyloid deposition in the epithelium and anterior stroma [14]. Additionally, three specimens initially treated with PKP (two diagnosed as lattice dystrophy and one as an unspecified epithelial-stromal dystrophy) were later reclassified as Gly623Asp mutation variants based on genomic DNA analysis from peripheral blood leukocytes. To ensure consistency within the dataset, these cases were retained under their original classifications of lattice dystrophy and unspecified epithelial-stromal dystrophy.

LimboKP was frequently utilized for GDLD (18/29) and for a significant subset of LCD (16/64) and GCD cases (20/79).

A single postmortem specimen was recorded as an incidental finding.

Further details on the distribution of surgical procedures for each dystrophy type are provided in Fig. 5.

## Discussion

This study provides a comprehensive, long-term analysis of histopathologically confirmed corneal dystrophies collected over eight decades. By evaluating trends in specimen distribution and surgical interventions, it contributes to the epidemiologic understanding of surgically relevant corneal dystrophies and offers a unique perspective on the evolution of clinical practice and surgical advancements at a tertiary referral center in Germany.

The study’s timeline was shaped by significant historical events. The destruction of the Eye Clinic and histology laboratory during World War II led to the complete loss of all samples and records before 1945. Although corneal samples were received and analyzed starting in 1945, the first specimen diagnosed as a corneal dystrophy was not recorded until 1966. This timing coincides with the histology laboratory being re-established in a new location under Wilhelm Wegner in 1964 and the appointment of Günter Mackensen as clinic director in 1967, who introduced microsurgical techniques. These events likely enabled the subsequent rise in corneal specimens submitted for analysis.

The distribution of dystrophy types revealed FECD as the most frequently identified dystrophy in surgical specimens, accounting for 90.3% of all cases. This aligns with findings by Spraul and Grossniklaus [15] (Atlanta, USA, 1941–1995), who reported that FECD constituted 92.6% of all corneal dystrophy specimens, even before the introduction of posterior lamellar keratoplasty.

A significant increase in FECD specimens was observed starting in the early 2000s, coinciding with the appointment of Thomas Reinhard as clinic director in 2003 and continuing with the subsequent widespread adoption of posterior lamellar keratoplasty techniques in the late 2000s. Since 2003, there has been a dramatic rise in FECD specimens, increasing from 15 cases in 2003 to 236 cases in 2024, representing a remarkable increase of over 15-fold. A notable decline in FECD specimen numbers occurred in 2009 and 2010, interrupting the otherwise steady upward trend. This drop likely reflects the delayed effects of the German Tissue Act (Gewebegesetz), implemented in 2007, which introduced stricter requirements for tissue donation, most notably the mandatory collection of donor blood samples within 24 h of death. The resulting complexity of the donation process necessitated a comprehensive reorganization of tissue procurement systems, which took approximately one to two years to implement, ultimately contributing to the temporary reduction in surgical procedures and corresponding specimen submissions during that period. Similarly, a temporary decline in specimen numbers was observed in 2020 and 2021, coinciding with the COVID-19 pandemic. This global health crisis led to a reduction in elective surgical procedures, including corneal transplantation [16], and likely contributed to the observed drop in FECD specimens during these years in this study. Following this decline, the marked rise in specimen numbers from 2022 to 2024 can be interpreted as a recovery phase, as clinical operations returned to full capacity and the backlog of surgeries delayed by the pandemic was addressed.

The surgical management of FECD at our center underwent a profound transformation. While posterior lamellar keratoplasty was first introduced in 2007 in our center, a pivotal tipping point was reached in 2011 when it surpassed traditional PKP for the first time, accounting for 54.4% of procedures. The adoption then accelerated dramatically, with posterior lamellar keratoplasty comprising 94.3% of all FECD surgeries just one year later in 2012. This new paradigm quickly solidified, and since 2017, posterior lamellar keratoplasty has consistently accounted for over 99% of surgeries for this condition. This trend mirrors the global shift towards minimally invasive endothelial keratoplasty techniques, which offer faster visual recovery, lower rejection rates, and improved long-term outcomes compared to traditional PKP [17]. However, the introduction of posterior lamellar keratoplasty complicates direct comparisons with studies conducted before its implementation, as it has likely influenced both the frequency and pattern of specimen submissions. The enhanced safety profile and improved outcomes of modern endothelial keratoplasty have almost certainly significantly lowered the clinical threshold for intervention, leading to a surge in surgical procedures for patients who would have been not been managed in the past. This profoundly complicates direct numerical comparisons with older histopathological studies from the PKP-only era. To our knowledge, our study is the first long-term analysis to document this transition and examine the proportional representation of FECD in the modern surgical era.

The dramatic rise in surgical interventions for FECD, documented in a well-supplied center, serves also to highlight the scale of the global challenge. This finding is particularly significant in the context of the global landscape described by Gain et al. [4], who identified FECD as the leading indication for transplantation (accounting for 39% of all corneal transplants in 2012) amidst a persistent and profound donor tissue shortage. This global imbalance underscores the urgency of improving tissue allocation strategies and expanding donation infrastructure tailored to regional needs, particularly as FECD continues to account for a substantial proportion of the global corneal transplant burden.

Our analysis of 372 non-Fuchs specimens revealed a broad heterogeneity, with granular, lattice, and macular dystrophies being the most prevalent. The proportion of unspecified granular dystrophy reflects the inability to differentiate between GCD1 and GCD2 in the earlier years of the study, as GCD2 (Avellino corneal dystrophy) was only first described by Folberg et al. [18] in 1988. This relatively balanced distribution among the three main types is broadly consistent with an earlier German series by Lang et al. [19].

This pattern, however, contrasts sharply with findings from other geographic regions, highlighting significant global variations as summarized in Table 2. For instance, LCD was a prominent finding in North American series, dominating the Canadian cohort by Santos et al. [7] and being the second most frequent diagnosis after EBMD in the historical US series by Spraul and Grossniklaus [15]. In sharp contrast, reports from Saudi Arabia consistently show an overwhelming majority of MCD cases, a finding attributed to regional genetic factors [20–22]. Similarly, while MCD was also common in a large series from South India by Pandrowala et al. [23], congenital hereditary endothelial dystrophy was unexpectedly the most frequent diagnosis, a phenomenon the authors linked to higher rates of consanguineous marriages [21, 23]. In yet another distinct pattern, the major series from Japan identified gelatinous drop-like dystrophy (GDLD) as its most common non-Fuchs dystrophy [24]. These regional differences highlight the importance of context-specific data. Comparable long-term histopathological studies from diverse populations are essential to better understand global variation and guide regionally adapted corneal care strategies.

**Table 2:** Comparison of Non-Fuchs dystrophy distribution in major histopathological series

Study (Region, Period)	Total (*n*)	Granular (GCD)	Lattice (LCD)	Macular (MCD)	Frequent ‘Other’ Dystrophy	Additional ‘Other’ Dystrophies
***Present Study (Germany***, ***1945–2024)***	*372*	***79 (21.2%)***	*64 (17.2%)*	*65 (17.5%)*	*GDLD (29*, *7.8%)*	*EBMD (25)*, *TBCD (15) and 13 other rarer types (see* Table 1*for full details)*
Lang et al. [19] (Germany, 1964–1985)	53	**20 (37.7%)**	10 (18.9%)	16 (30.2%)	CHED (3)	EBMD (2), PPCD (1), RBCD (1)
Spraul & Grossniklaus [15] (USA, 1941-95)	126	16 (12.7%)	29 (23.0%)	18 (14.3%)	**EBMD (53**, **42.1%)**	PPCD (7), CHED (1), SCD (1)
Santos et al. [7] (Canada, 1996–2006)	26	8 (30.8%)	**17 (65.4%)**	1 (3.8%)	None	-
Al Faran et al. [20] (Saudi Arabia, 1983-88)	74	3 (4.1%)	5 (6.8%)	**53 (71.6%)**	CHED (11)	RBCD (2)
Alzuhairy et al. [21] (Saudi Arabia, 2002-11)	193	9 (4.7%)	4 (2.1%)	**180 (93.3%)**	None	-
Kokandi et al. [22] (Saudi Arabia, 2015-19)	22	0 (0%)	0 (0%)	**22 (100%)**	None	-
Pandrowala et al. [23] (South India, 1995-01)	151	4 (2.6%)	15 (9.9%)	53 (35.1%)	**CHED (63**, **41.7%)**	GDLD (8), PPCD (5), RBCD (3)
Santo et al. [24] (Japan, 1959-92)	158	44 (27.8%)	38 (24.1%)	21 (13.3%)	**GDLD (47**, **29.7%)**	CHED (2), PPCD (2), SCD (2)

Percentages represent the proportion of the total number of non-Fuchs specimens for each series. Bold indicates the most frequent dystrophy for each series

CHED, Congenital hereditary endothelial dystrophy; EBMD, Epithelial basement membrane dystrophy; GCD, Granular corneal dystrophy; GDLD, Gelatinous drop-like corneal dystrophy; LCD, Lattice corneal dystrophy; MCD, Macular corneal dystrophy; PPCD, Posterior polymorphous corneal dystrophy; RBCD, Reis–Bücklers corneal dystrophy; SCD, Schnyder corneal dystrophy; TBCD, Thiel–Behnke corneal dystrophy

This study, in addition to being more contemporary, spans a significantly longer time period than others and remarkably encompasses nearly all dystrophies categorized under the IC3D classification. However, while cases of Franceschetti Dystrophy were identified in this collective, no cases of Dystrophia Smolandiensis (DS) or Dystrophia Helsinglandica (DH), other subtypes of Epithelial Recurrent Erosion Dystrophies, were found. This absence aligns with prior discussions in the literature questioning the distinctiveness of these entities as separate classifications [3]. Additionally, the lack of histopathological samples from Fleck Corneal Dystrophy (FCD) and Central Cloudy Dystrophy of François (CCDF) is consistent with the clinical characteristics of these dystrophies, which are typically asymptomatic and rarely necessitate surgical intervention [3]. In the endothelial category, no cases of X-linked Endothelial Corneal Dystrophy (XECD) were observed. This could be attributed to its extreme rarity or the possibility that such cases were included within the broader category of unspecified endothelial dystrophies, where histopathological features were insufficiently distinct for precise classification.

The surgical management of non-Fuchs corneal dystrophies in this study highlights the diversity of approaches necessitated by the heterogeneity of these conditions. Surgical techniques varied significantly based on anatomical localization, severity, and pathological mechanisms, reflecting advancements in corneal surgery and the growing emphasis on pathology-specific interventions.

PKP remained the predominant surgical method for stromal dystrophies throughout the analyzed period, particularly for deeply diffuse located deposits in MCD (64/65 specimens), LCD (46/64), and GCD (38/79). This aligns with other studies that identified PKP as the primary approach for stromal and epithelial-stromal dystrophies. Pandrowala et al. [23] and Santos et al. [7] reported the exclusive use of PKP, while Lang et al. [19] and Santo et al. [24] mentioned the use of lamellar keratoplasty and keratectomy, but without specifying numbers. In contrast, Alzuhairy et al. [21] found that 10 of 180 MCD cases were treated with lamellar keratoplasty, with the remainder undergoing PKP. Notably, deep anterior lamellar keratoplasty (DALK) was similarly not utilized for any dystrophies in this series. This suggests a widespread and persistent view of PKP as the definitive treatment for these conditions, likely to ensure the complete removal of all diseased tissue and potentially minimize long-term recurrence.

A notable shift in surgical practice in this series occurred after 2003 with the introduction of LimboKP by clinic director Thomas Reinhard for dystrophies at high risk of recurrence, such as GDLD, LCD, and GCD. By transplanting limbal stem cells, the donor cornea was trephined to include a segment containing the donor limbus, which was then centrally sutured into the host cornea [25, 26]. LimboKP aimed to reduce recurrence rates, particularly in dystrophies of epithelial origin [26, 27].

For superficial dystrophies, tailored surgical approaches such as MSK and PTK became prominent in managing conditions involving the epithelial or anterior stromal layers. MSK was the preferred choice for epithelial dystrophies, including Lisch Epithelial Corneal Dystrophy and Epithelial Basement Membrane Dystrophy. PTK, on the other hand, was primarily employed for anterior stromal dystrophies, such as granular dystrophies, Thiel-Behnke Dystrophy and Reis-Bückler’s Dystrophy, reflecting approaches reported in the literature [10, 28].

Although endothelial dystrophies were less common in the non-Fuchs category, they were predominantly managed with PKP, as DMEK only became widely available and adopted in more recent years. In later cases, DMEK was selectively employed, reflecting its emerging role in managing endothelial dystrophies and its potential as a minimally invasive alternative to PKP, as reported in the literature [29–31]. These trends illustrate the evolution of surgical strategies over time, highlighting a shift toward less invasive, more targeted approaches that precisely address disease-specific pathologies.

This study has limitations that warrant consideration. Its retrospective design introduces biases, including potential confounding factors and incomplete clinical data. As a single-center analysis, the findings may not be generalizable to populations with differing genetic backgrounds, healthcare systems, or clinical practices. Since corneal dystrophies do not always require surgery, focusing solely on surgical specimens underestimates their true prevalence. Nevertheless, the findings provide a valuable indicator of the frequency and demographic characteristics of cases severe enough to necessitate surgical intervention. The number of specimens is also influenced by surgical recurrences, which may inflate case counts for certain dystrophies. Investigating recurrence patterns in future studies could therefore yield important additional insights. The nearly eight-decade timeline introduces variability due to evolving diagnostic criteria, methods, and nomenclature, which may impact the consistency of the data. Additionally, while the difference between disease onset and surgical age limits insights into the natural progression of dystrophies, it offers valuable information on when these conditions progress to a stage requiring surgical intervention.

However, the foundation of this study on a comprehensive pathology archive provides unique strengths. It ensures data longevity and consistency across eight decades, a scope not readily achievable with clinical records due to their usually shorter retention periods and heterogeneous systems. Furthermore, histopathological examination offers a gold-standard for diagnostic certainty, allowing for robust classification according to modern nomenclature and correcting for known discrepancies between initial clinical diagnoses and definitive pathological findings. Finally, as our laboratory serves as a major referral center for Southwest Germany, the dataset affords a comprehensive regional perspective on the burden of surgically-managed dystrophies, extending beyond that of a single institution.

## Conclusions

To our knowledge, this is the only single-center analysis to date that encompasses nearly all IC3D-classified dystrophies within a single histopathological dataset. This study provides valuable insights into the distribution, demographics, and surgical trends of corneal dystrophies over eight decades, highlighting the predominance and sharp increase of surgically-treated FECD cases, alongside the shift toward posterior lamellar keratoplasty. In contrast, non-Fuchs dystrophies exhibited great heterogeneity in both clinical presentation and surgical management. Granular, lattice, and macular dystrophies emerged as the most frequently encountered types. Surgical approaches varied accordingly: minimally invasive procedures such as MSK and PTK were employed for superficial dystrophies, while limbo-keratoplasty was introduced for epithelial-stromal forms with high recurrence risk. These developments reflect a broader shift from conventional full-thickness grafts toward more personalized, pathology-specific surgical strategies, underscoring the ongoing need for management approaches tailored to disease characteristics and epidemiologic profiles of each population. Given the marked geographic variability in dystrophy type prevalence, generating comparable longitudinal datasets from other regions is particularly valuable to help guide regional and global eye care policy.

## Data Availability

The datasets used and analyzed during the current study are available from the corresponding author upon reasonable request.

## Abbreviations

CHED: Congenital hereditary endothelial dystrophy
CCDF: Central Cloudy Dystrophy of François
CSCD: Congenital stromal corneal dystrophy
DH: Dystrophia Helsinglandica
DMEK: Descemet membrane endothelial keratoplasty
DS: Dystrophia Smolandiensis
DALK: Deep Anterior Lamellar Keratoplasty
DSAEK: Descemet Stripping Automated Endothelial Keratoplasty
EBMD: Epithelial basement membrane dystrophy
ERED: Epithelial recurrent erosion dystrophy
FCD: Fleck Corneal Dystrophy
FECD: Fuchs endothelial corneal dystrophy
FRCD: Franceschetti corneal dystrophy
GCD: Granular corneal dystrophy
GCD1: Granular Corneal Dystrophy Type 1
GCD2: Granular Corneal Dystrophy Type 2
GDLD: Gelatinous drop–like corneal dystrophy
H&E: Hematoxylin and eosin
IC3D: International Committee for the Classification of Corneal Dystrophies
IQR: Interquartile Range
LCD: Lattice corneal dystrophy
LECD: Lisch epithelial corneal dystrophy
LimboKP: Limbo–keratoplasty
MCD: Macular corneal dystrophy
MECD: Meesmann corneal dystrophy
MSK: Manual superficial keratectomy
NFD: Non–Fuchs dystrophies
PACD: Posterior amorphous corneal dystrophy
PAS: Periodic acid–Schiff
PDCD: Pre–Descemet corneal dystrophy
PKP: Penetrating keratoplasty
PPCD: Posterior polymorphous corneal dystrophy
PTK: Phototherapeutic keratectomy
RBCD: Reis–Bücklers corneal dystrophy
SCD: Schnyder corneal dystrophy
SMCD: Subepithelial mucinous corneal dystrophy
TBCD: Thiel–Behnke corneal dystrophy
TGFBI: Transforming growth factor, beta–induced
XECD: X–linked endothelial corneal dystrophy
